# A Monte Carlo approach to estimate the uncertainty in soil CO_2_ emissions caused by spatial and sample size variability

**DOI:** 10.1002/ece3.1729

**Published:** 2015-09-23

**Authors:** Wei‐Yu Shi, Li‐Jun Su, Yi Song, Ming‐Guo Ma, Sheng Du

**Affiliations:** ^1^ State Key Laboratory of Loess and Quaternary GeologyInstitute of Earth EnvironmentChinese Academy of SciencesXi'anShaanxi710061China; ^2^ State Key Laboratory of Soil Erosion and Dryland Farming on Loess PlateauInstitute of Soil and Water ConservationNorthwest A&F UniversityYanglingShaanxi712100China; ^3^ School of ScienceXi'an University of TechnologyXi'anShaanxi710054China; ^4^ Cold and Arid Regions Environmental and Engineering Research InstituteChinese Academy of SciencesLanzhou730000China

**Keywords:** Maize, Monte Carlo approach, oasis, soil respiration, uncertainty

## Abstract

The soil CO_2_ emission is recognized as one of the largest fluxes in the global carbon cycle. Small errors in its estimation can result in large uncertainties and have important consequences for climate model predictions. Monte Carlo approach is efficient for estimating and reducing spatial scale sampling errors. However, that has not been used in soil CO_2_ emission studies. Here, soil respiration data from 51 PVC collars were measured within farmland cultivated by maize covering 25 km^2^ during the growing season. Based on Monte Carlo approach, optimal sample sizes of soil temperature, soil moisture, and soil CO_2_ emission were determined. And models of soil respiration can be effectively assessed: Soil temperature model is the most effective model to increasing accuracy among three models. The study demonstrated that Monte Carlo approach may improve soil respiration accuracy with limited sample size. That will be valuable for reducing uncertainties of global carbon cycle.

## Introduction

The total global emission of CO_2_ from soils is recognized as one of the largest fluxes in the global carbon cycle (Schlesinger and Andrews 2000; Piao et al. 2009; Bond‐Lamberty and Thomson 2010) and plays a major role in determining the atmospheric greenhouse effect (Field et al. 2007). This large annual emission dwarfs anthropogenic CO_2_ production from fossil fuel and implies that any small error in its estimation would result in large uncertainties related to the effects of CO_2_ build‐up in the atmosphere. There is a need, therefore, to improve the accuracy of soil CO_2_ emission estimates (Shi et al. 2012; Gomez‐Casanovas et al. 2013).

Actually, numerous studies regarding the uncertainties of carbon flux estimates using eddy covariance (EC) have been reported. Elbers et al. (2011) presented a method for evaluating the factors of total uncertainty for estimating net ecosystem productivity (NEP) without considering spatial variability. Richardson and Hollinger (2007) used synthetic data sets, developed by assimilating data from a range of FLUXNET sites, into a simple ecosystem model to evaluate the relationship between gap length and uncertainty in the net ecosystem exchange (NEE) of CO_2_. Oren et al. (2006) assessed spatial variability estimates in the context of uncertainty in the annual NEE and combined uncertainty from gap filling and instrument error with the uncertainty caused by spatial variability. Hou et al. (2013) evaluated the effects of the spatial heterogeneity of reservoir permeability on CO_2_ migration, applying an uncertainty quantification framework. However, all above studies focus mainly on eddy covariance (EC) methods (e.g., NEE, NEP, and ecosystem respiration), and uncertainty in soil respiration estimation remains far greater than that in other components of the carbon cycle (Bond‐Lamberty et al. 2004; Trumbore 2006; Zhang et al. 2013). As with the ecosystem carbon cycle, the uncertainty of soil respiration also contains measurement error and flux calculation uncertainty, spatial variability uncertainty, statistical selection uncertainty, and gap‐filling uncertainty.

Methods for measuring soil CO_2_ efflux have undergone a considerable evolution over the past 30 years, giving rise to what is today considered state‐of‐the‐art measuring systems that consist of automated chambers that use an infrared gas analyzer (Pumpanen et al. 2004; Subke et al. 2006; Vargas et al. 2011; Jassal et al. 2012; Koskinen et al. 2014; Maier and Schack‐Kirchner 2014; Riederer et al. 2014). These systems are often deployed as nonsteady state or steady state. Static chamber systems also continue to play an important role in assessing soil CO_2_ emissions because they are relatively inexpensive and easy to deploy. These measurement systems are generally considered to provide the most reliable estimates we have of soil respiration. (Shi et al. 2011; Maier and Schack‐Kirchner 2014). Sources of uncertainties in soil respiration stem from site characterization, site carbon capacity, injection rate, CO_2_ trapping mechanisms, mineral precipitation dissolution kinetics, and so on (Hou et al. 2013). In general, all sources of uncertainty can be divided into two dimensions: time and space. It is well recognized that static chambers have poor temporal resolution and automated chambers have poor spatial resolution in these dimensions.

To address the problem of temporal uncertainty, gap‐filling strategies have been applied effectively to estimate the soil CO_2_ efflux, and the soil respiration estimating model has been assessed according to time (Gomez‐Casanovas et al. 2013); however, the study only focused on time‐series sampling, and spatial uncertainty has not been investigated. For spatial uncertainty, the inventory method is limited by the quality and the spatiotemporal representativeness of measured Rs data. Poor data can result in infinite uncertainty on Rs estimates on a regional scale (Yu et al. 2010). Furthermore, the process‐based soil respiration model has always been considered as the universal method for both temporal and spatial estimation of soil respiration. This method can simulate the spatial patterns and also predict the long‐term dynamics of ecosystem respiration (Cramer et al. 2001). However, the process‐based soil respiration model has a complicated structure when connecting soil–plant–atmosphere processes. It is thus difficult to evaluate the rationality of the estimated results when considerably large uncertainty exists in the spatial representativeness of model parameters (Yu et al. 2010). Nevertheless, compared with the above methods, the geostatistical model of soil respiration could be a good method due to simple structure, sound parameterization method, and reasonable results (Raich and Potter 1995; Reichstein et al. 2003), but the application of this method is built on the premise that relationships exist between in situ soil respiration and environmental variables. However, in natural conditions, randomness is universal and authentic, especially for temporal variation.

Monte Carlo method complies with this natural randomness and only relies on sufficient data and repeated random sampling, without considering any premise. These methods are most suited to calculation by a computer and tend to be used when it is infeasible to compute an exact result with a deterministic algorithm. Additionally, the method is used to complement derivations (Doucet et al. 2000). Currently, the Monte Carlo sampling technique is an efficient method for estimating and then reducing spatial‐scale sampling error. It has been applied for estimating transpiration (*E*) of forest stands (Kumagai et al. 2005a) and for examining how errors in *E* would be generated from different parameter values acquired with an equation regressed with limited data (Kumagai et al. 2005b; Kume et al. 2010). However, the Monte Carlo sampling technique has not been used in other similar fields, for example, soil CO_2_ emission estimation. The technique may eventually play an irreplaceable role in this estimation.

In this study, we aimed to define an optimal and effective sampling design to determine 10s km‐scale soil CO_2_ emission estimates calculated from soil respiration rate measurements, examine how sample sizes for soil temperature and soil moisture impact these 10s km‐scale soil CO_2_ emission estimates, determine whether the estimation errors due to sample sizes change with the variations in region area, and then build a standard Monte Carlo sampling procedure for producing defensible estimates of soil CO_2_ emission. Based on the assumption that the 10s km‐scale soil CO_2_ emission was accurately determined from point measurements, the impact of point‐to‐point variations in soil temperature and soil moisture on the 10s km‐scale soil CO_2_ emission will also be determined using a Monte Carlo analysis of the original data sets. This analysis predicted how many samples are required to account for point‐to‐point variations and evaluated the applicability of three general soil CO_2_ emission estimating models.

## Materials and Methods

### Study site

The study site is located in the Zhangye oasis (1,400–1,600 m a.s.l.), Gansu Province, China, which is the core part of the middle reaches of the Heihe River. The climate is temperate, with a mean annual temperature of 7.6°C, mean annual precipitation of 117 mm, and mean annual potential evaporation of 2390 mm (Wang et al. 2013). The main crops cultivated in this area are maize and wheat. Almost all farmland in this area is irrigated with the water diverted from the Heihe River. The field observations used in this study were derived from the Heihe Watershed Allied Telemetry Experimental Research (HiWATER) project. HiWATER is a comprehensive eco‐hydrological experiment under the framework of the Heihe Plan and is based on the diverse needs of interdisciplinary research and existing observational infrastructures in the basin (Li et al. 2013).

A permanent observation plot was set up in the farmland, which is located within 38.8369°‐38.9055°N, 100.332°‐100.410°E, covering an area of 5.0 × 5.0 km^2^ and is cultivated with maize (*Zea mays L*.) during the experimental period. Fifty‐one custom‐designed polyvinyl chloride (PVC) collars were placed in the observation plot for measurements of CO_2_ efflux from the soil. The plot was equally divided into four 2.5 × 2.5 km^2^ subplots. Different numbers of PVC collars (observation points) were evenly established in each subplot. Detailed information of the different plot layouts is shown in Table S1.

### Measurement of soil CO_2_ efflux, soil temperature, and moisture

Soil respiration was measured using an automated soil CO_2_ flux system (LI‐8100; LI‐COR, Lincoln, NE, USA) equipped with a portable chamber (Model 8100‐103). A PVC collar (20.3 cm in diameter and 10 cm in height) was inserted into the soil among the maize seedlings to a depth of 2.5 cm at each sampling point approximately 2 weeks before the first measurement. Small litter was left in the collar, and large items were removed. All collars were left at the site for the entire study period.

The soil respiration data from the 51 PVC collars were measured once every 6 days over the whole period of maize growth from 6 June to 19 September 2012. The maize in the study site was harvested at approximately this time. Based on the preliminary experiment in 2011 (continuous measurement of soil respiration), the suitable diurnal measurement time was determined: Measurements were taken between 8:30 and 12:00 local time on each sampling day. Preliminary experiment in detail was described in Appendix S1.

Temporal soil temperature and moisture near each collar were measured at the same time as soil respiration measurements. Soil temperature was measured at a depth of 10 cm using a handle thermocouple probe, while the soil volumetric water content was measured at 0–10 cm depth, using a time‐domain reflectometry moisture meter (TDR200; Spectrum, Aurora, IL, USA).

Similarly, continuous soil temperature and moisture near each collar were also measured throughout the entire study period. Soil temperature was measured at 10 cm depth by thermorecorders (TR‐52; T&D, Matsumoto, Japan), and soil moisture was measured at 10 cm by a soil moisture sensor (SMB‐M005; Decagon Devices, Pullman, WA, USA). The continuous measurements were performed at 30‐s intervals, and 30‐min averages were recorded.

### Scaling for growing seasonal soil CO_2_ efflux

Soil respiration data from the complete growing season were fitted to soil temperature and water content with exponential and power functions given in equations (1) and (2) to describe the dependence of soil respiration on soil temperature and soil water content.

(1) $$R=\alpha \times e^{\beta T}$$

(2) $$R=\alpha \times W^{\beta }$$

where *R*,* T*, and *W* are soil respiration, soil temperature, and soil volumetric water content, respectively, and *α* and *β* are constant coefficients. An equation with two variables was established to describe the interactive effects of soil temperature and water content on soil respiration (Li et al. 2008a):

(3) $$R=\alpha \times T^{\beta }\times W^{\gamma }$$

where *α*,* β*, and *γ* are constant coefficients.

A soil CO_2_ emission of 51 points over the growing season was calculated by integrating the CO_2_ efflux for the period from 16 June to 19 September 2012 using the observed ecosystem‐specific response equations: (1), (2), and (3). Applicability of this method has been demonstrated by some studies (Wang et al. 2010b; Shi et al. 2014). Equations (1), (2), and (3) were abbreviated as *R*:*T*,* R*:*W*, and *R*:*T*&*W*, respectively.

Furthermore, the coefficients of determination (*R* ^2^) of different models on soil respiration (*R*) against soil temperature (*T*) and/or soil moisture (*W*) were calculated by nonlinear least‐squares method. The results were from average coefficients of determination (*R* ^2^) value of 51 points.

### Method of analysis

The soil CO_2_ emission of every point was calculated by, respectively using equations (1), (2), and (3). Besides, *W* and *T* of the every point were represented by their average value. The estimation errors of *R*,* W*, and *T* caused by spatial variations were calculated using various sample size based on Monte Carlo sampling.

**Figure 1:**
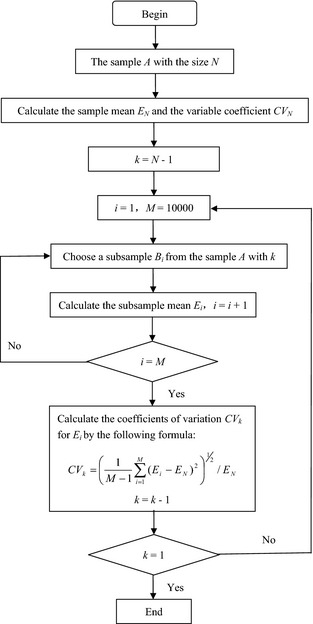
Diagram of the Monte Carlo sampling computer program.

Figure 1 shows the flow diagram of the Monte Carlo sampling computer program, and the specific steps are shown as follows:

1 The size of a sample *A* is *N (N *=* *51; respectively for *R*,* W* and *T)*, and the arithmetic mean value *E*_*N*_ of this sample was calculated.
2 The subsamples *B*_*i*_ with the sample size *k *= *N* – 1 were chosen from the sample about *M *=* *10,000 times, randomly. The arithmetic mean values *E*_*i*_ of each subsample *B*_*i*_ were calculated and *i *=* *1, 2, …, *M*. The sampling for subsamples *B*_*i*_ is a random process, and it can be thought that the subsamples are different, because it was decided by the sampling method. We assume the sample *A *= [*A*_1_, *A*_2_,…, *A*_N_], and the method is shown as follows:
  - *N* is the size of a sample *A*, and the numbers from 1 to N were rearranged randomly. The rearranging result *C*_1_ was a vector, for example, *C*_1_ *= a*_1_, *a*_2_,…, *a*_*N*_ (*a*_*i*_ = 1, 2,…, *N*,* a*_*i*_ ≠ *a*_*j*_).
  - The other vector *D* was obtained by choosing numbers from *C*_1_ before the number *k*. Thus, *D*_1_ = *a*_1_, *a*_2_,…, *a*_*k*_;
  - The subsamples *B*_1_ were obtained by choosing the data *A*_*ai*_(*i *=* *1, 2, …, *k*), so $B_{1}=\left[A_{a1},A_{a2},\ldots ,A_{ak}\right]$;
  - The steps a–c were repeated 10000 times, and the vector groups *C*_*i*_, *D*_*i*_ and *B*_*i*_ (i=1,2,…,10,000) were obtained. Because the rearranging process for *C*_*i*_ was random, $C_{i}=C_{j}\left(i,j=1,2,\ldots ,10,000,i\neq j\right)$ was a little probability event. There were few of *D*_*i*_ = *D*_*j*_ or $B_{i}=B_{j}\left(i,j=1,2,\ldots ,10,000,i\neq j\right)$, but compared with 10000 groups data, the effect on the results can be ignored.

3 *E*_*N*_ was used as the arithmetic mean value of the subsample *B*_*i*_ mean, *E*_*i*_. Thus, the coefficients of variation CV_*k*_ for *E*_*i*_ with the sample size *k* can be calculated by the following formula:

$${\text{CV}}_{k}={\left(\frac{1}{M-1}\sum_{i\;=\;1}^{M}{\left(E_{i}-E_{N}\right)}^{2}\right)}^{1/2}/E_{N}$$

When *M* is large enough, based on the law of large numbers, CV_*k*_ means the degree of variation for the subsample with measure times *k* is compared with the sample with measure times *N*.

4 Let *k *= *k* – 1, and repeat steps 2–3. Calculate the coefficients of variation (CV_*k*_) of the subsample mean with the sample size *k* and *k *=* *1, 2…, N‐1.

Additionally, In this study, the equations (1), (2), and (3), respectively, assume that the variance of *R* estimates associated with sample sizes was given by variance of *T* estimates, variance of *W* estimates and combined variance of *T*&*W* estimates associated with sample size. Also, constant coefficients in equations (1), (2), and (3) were determined by the relation of *R* and *T* and/or *W* to potential estimation errors. The total derivative of the equations (1), (2), and (3) is as follows:

(4) $$dR=\alpha \cdot \beta \cdot e^{\beta T}dT$$

(5) $$dR=\alpha \cdot \beta \cdot W^{\beta -1}dW$$

(6) $$dR=\alpha \cdot \beta \cdot W^{\gamma }\cdot T^{\beta -1}dT+\alpha \cdot \gamma \cdot T^{\beta }\cdot W^{\gamma -1}dw$$

The form of the equations (1), (2), and (3) can be transformed to the following equation:

(7) $$\frac{\Delta R}{R}=\beta \Delta T$$

(8) $$\frac{\Delta R}{R}=\beta \frac{\Delta W}{W}$$

(9) $$\frac{\Delta R}{R}=\beta \frac{\Delta T}{T}+\gamma \frac{\Delta W}{W}$$

Equations (7), (8), and (9), respectively, mathematically indicated sources of errors from the three estimation models: the equation (7) means the errors in *R* caused by the potential estimation variation in *T* associated with the sample size; the equation (8) means the errors in *R* caused by the potential estimation errors in *W* associated with the sample size; and the equation (9) means the errors in *R* caused by the potential estimation errors in *T* and *W* associated with the sample size; The analyses were performed using data sets collected from continuous measurements for *T* and *W*. The similar mathematical deduction method has been effectively applied for estimation of tree stand‐scale transpiration (Kumagai et al. 2005a,b; Kume et al. 2010).

To examine whether the potential errors due to sample sizes change in different plot conditions, Monte Carlo analyses were performed for subplots with different point densities (i.e., four 2.5 × 2.5 km^2^ subplots and four 5 × 2.5 km^2^ subplots)

## Results

### Variation in soil respiration

**Figure 2:**
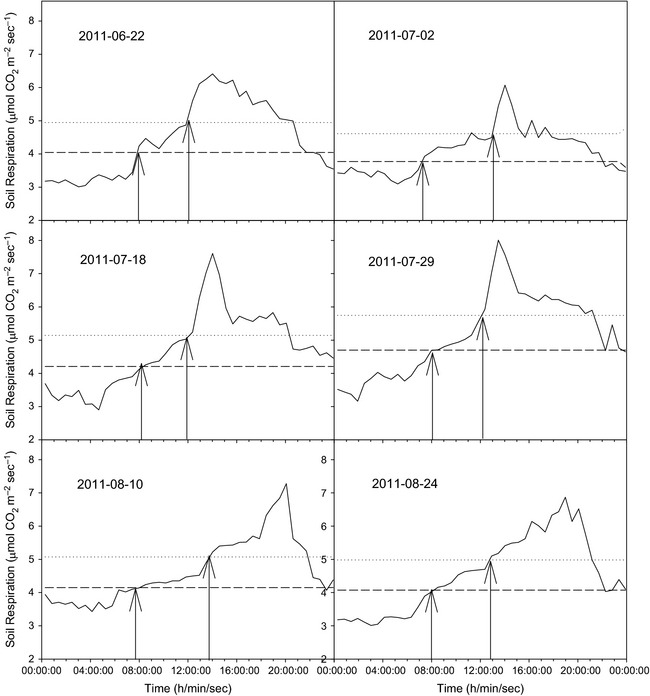
Soil respiration was automatically continuously measured in a preliminary experiment from 19 June to 31 August 2011. The general diurnal pattern was determined from different typical days in this period. The dash line represents the upper and lower bounds of the daily average value of soil respiration (diurnal average value ± 10% error), and the arrow points to the appearance of these boundaries.

The general pattern of the change in soil respiration during every day of the preliminary experimental period was similar (preliminary experimental design in detail see appendix S1). There was a strong diurnal pattern with a peak in the period. Figure 2 shows the typical diurnal pattern of soil respiration in representative day of this period. In this period, the diurnal average value is approximately 4.5 *μ*mol·m^−2^·s^−1^, and the diurnal peak value is 7.0 *μ*mol·m^−2^·s^−1^. Nevertheless, the time of the peak value occurring was not steady, but the diurnal average value ± error was located between approximately 7:30 and 12:30. Additionally, the maximum error is defined as 10% of the average value.

### Sample size

**Figure 3:**
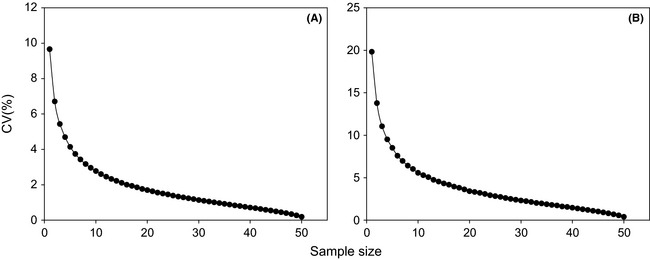
Application of the spatial Monte Carlo technique. (A) Relationship between sample size and CV in *T*; (B) relationship between sample size and CV in *W*.

Figure 3 shows the relation between the sample size and the CV of *T* & *W* in the 5 × 5 km^2^ plot. Two‐dimensional analytic geometry has demonstrated that if the slope of the curve is less than −1.0, the value of the vertical axis will change more slowly compared with abscissa axis; if more than −1.0, the situation will be reversed. Therefore, −1.0 for dCV/d*n* is deemed as the threshold of significant changes of CV (dCV/d*n*) for estimating the optimal sample size. A dCV/d*n* < −1.0 indicates that the CV significantly decreased and greatly improved the precision of the estimation with an increase in the number (*n)* of PVC collars. In contrast, a dCV/d*n* > −1.0 suggests a slight decrease with increasing *n*, and the increase of *n* cannot effectively improve the estimation precision. In this study, the minimum n at dCV/d*n* > −1.0 is defined as the optimal sample size. According to this threshold value, *n *=* *3 for the soil temperature was the optimal sample size, and the CV was 5.4%. When *n* was less than 3, the dCV/d*n* for the soil temperature was smaller than −1.0. Conversely, when *n* was more than 3, the dCV/d*n* for soil temperature was greater than −1.0 (Fig. 3A). Similarly, the dCV/d*n* for the soil moisture was larger than −1.0 at *n *>* *4, and the dCV/d*n* for the soil moisture was smaller than −1.0 at *n *<* *4 (Fig. 3B). The optimal sample size for the soil moisture was *n *=* *4 and CV was 9.5.

The optimal sample size of *T*,* W*, and *E* estimates with *R*:*T*,* R*:*W*, and *R*:*T*&*W* was analyzed by the Monte Carlo method (Table 1). Depending on the threshold value (dCV/d*n* = −1.0), different optimal sample sizes were indicated for *T*,* W*, and *E* in one 5.0 × 5.0 km^2^ plot and four 5.0 × 2.5 km^2^ plot. Furthermore, in four 2.5 × 2.5 km^2^ plots, the optimal sample size was not obtained because all dCV/d*n* were smaller than −1.0.

**Table 1:** Optimal sample sizes for estimating *T*,* W*, and *R*, based on three models in the 5.0 × 5.0 km^2^ plot, consisting of four 2.5 × 2.5 km^2^ subplots. Potential errors were determined from the Monte Carlo method

	Point numbers	*T*		*W*		*R* = *α* × *e* ^*βT*^		*R* = *α* × *W* ^*β*^		*R* = *α* × *T* ^*β*^ × *W* ^*γ*^	
		*n*	CV (%)	N	CV (%)	N	CV (%)	N	CV (%)	*n*	CV (%)
5.0 × 5.0 km2 plot	51	3	5.4	4	9.5	6	11.3	6	11.4	6	10.8
5.0 × 2.5 km^2^ subplot											
Subplot 1 + 2	30	3	6.3	6	8.5	7	11.2	6	10.6	6	10.7
Subplot 2 + 3	27	3	6.2	6	8.6	7	11.5	7	10.6	7	10.0
Subplot 1 + 4	24	3	9.5	6	9.0	6	10.6	7	9.2	6	9.2
Subplot 3 + 4	21	2	2.1	4	6.1	7	8.6	7	9.0	6	9.1

### Variation of errors in a different method for estimating *E*

**Figure 4:**
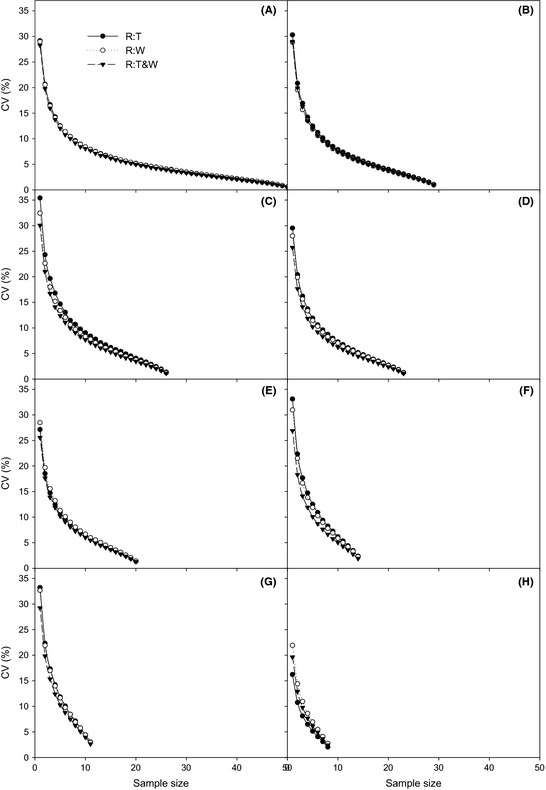
Relationship between sample size and CV in *R* estimated by *R*:*T* (closed circle), *R*:*W* (open circle), and *R*:*T*&*W* (closed triangle) models for different point numbers: 51 (A), 30 (B), 27(C), 24 (D), 21 (E), 15(F), 12 (G), and 9 (H) applying Monte Carlo method.

Figure 4 shows the variation of errors in *E* estimated by *R*:*T*,* R*:*W*, and *R*:*T*&*W*, respectively, associated with the sample size in different plots. There are no significant differences between *R*:*T*,* R*:*W*, and *R*:*T*&*W* for 51 points in the 5.0 × 5.0 km^2^ plot and for 30 points in the 5.0 × 2.5 km^2^ plot. The dCV/d*n* of *E* estimates by *R*:*T*&*W* was larger than with the other two models, with *n* increasing for 27, 24, and 21 points in the 5.0 × 2.5 km^2^ plot and for 15 and 12 points in the 2.5 × 2.5 km^2^ plot. However, for 9 points in the 2.5 × 2.5 km^2^, the dCV/d*n* of the *E* estimates by *R*:*T* was larger than for *R*:*W* and *R*:*T*&*W*, with *n* increasing.

### Constant coefficient of errors in different estimate methods

Constant coefficients of three different methods were obtained by the total derivative of equations (1), (2), and (3). The equations (7), (8), and (9) indicated the influence of *T* and/or *W* on the error in *R*. Table 2 shows the constant coefficient in the different estimate methods for different size samples. The *β* of *R*:*T* is approximately 0.15, except for point numbers (points number/area) 21 (1.68 n·km^−2^) and 12 (1.92 n·km^−2^). And the *β* of *R*:*T* is approximately 1.3; nevertheless, the *β* value is higher in point numbers (points number/area) 21 (1.68 n·km^−2^), 12 (1.92 n·km^−2^), and 9 (1.44 n·km^−2^). Furthermore, the *β* and *γ* of *R*:*T*&*W* are not all positive, due to the interaction between *T* and *W*.

**Table 2:** Constant coefficient of errors with point number changes in the *R*:*T*,* R*:*W*, and *R*:*T*&*W* models of soil CO_2_ emission estimation

Point numbers	Points number/area (n·km^−2^)	*R*:*T* a	*R*:*W*	*R*:*T*&*W*	
		*β*	*β*	*β*	*γ*
51	2.04	0.154	1.473	1.649	0.622
30	2.4	0.131	1.259	0.833	0.834
27	2.16	0.162	1.387	0.05	1.261
24	1.92	0.13	1.226	2.401	−0.075
21	1.68	0.479	2.161	4.422	0.943
15	2.4	0.122	1.088	1.314	0.318
12	1.92	0.714	2.153	11.423	0.064
9	1.44	0.273	2.677	−1.436	2.935

a All regressions were significant at *P *<* *0.001.

## Discussion

In this study, the measurements were taken between 8:30 and 12:00 local time on each sampling day. More and more studies are reporting soil respiration over a time range during the day to better represent daily average values (Huang et al. 2007; Li et al. 2008b; Wang et al. 2010a). However, because environments are not different, the typical time ranges are also different. In a preliminary experiment, we confirmed that the diurnal average value ± error (10%) was about located between 7:30 and 12:30 (Fig. 2). In this time range and 5 × 5 km^2^ area, observation points maximally reached 51. Furthermore, number and space representations are key for Monte Carlo spatial sampling. These points were evenly distributed in consideration of the representative and experimental situation (Table S1). To a large extent, soil CO_2_ emission at this site was accurately measured, and choosing new points did not result in significant changes. Therefore, the number and location of these points are reasonable for the new sampling technique promotion.

There are mainly two sources of the error resulting from the estimation of the model parameter(s) and the integrating of the model prediction for *R*. In fact, this kind of error was from fitting degree of the three models on soil respiration (*R*) against soil temperature (*T*) and/or soil moisture (*W*). Table 3 indicated the uncertainty from the two sources by analyzing coefficients of determination (*R* ^2^). In our original application of the Monte Carlo method, we analyzed the optimal sample sizes and potential errors (CV) by evaluating the dCV/d*n* value, based on a data set with sample size changes and with variation in stand condition. In the 2.5 × 2.5 km^2^ subplot, the optimal sample sizes for *R*,* T*, and *W* were not obtained; this suggests that the sample sizes in the area were too small to significantly reduce the error (Fig. 4). Nevertheless, the ranges of the optimal samples sizes and the potential errors in the 5 × 2.5 km^2^ subplots were similar to those in the 5 × 5 km^2^ plot (Table 1). The results indicate that a small plot with a large enough sample size also can introduce a similar optimal error based on optimal sample size.

**Table 3:** Comparison of coefficients of determination (*R* ^2^) of three models on soil respiration (*R*) against soil temperature (*T*) and/or soil moisture (*W*)

Models^a^	Coefficients of determination (*R* ^2^ ± SE^b^)
*R*:*T*	0.283 ± 0.116
*R*:*W*	0.556 ± 0.132
*R*:*T*&*W*	0.692 ± 0.147

a All regressions were significant at *P *<* *0.001.

b SE: stander error, which is from 51 points.

*T* and *W* are two dominant factors for soil respiration from homogenous soil and vegetation (Xu and Qi 2001; Shi et al. 2012; Dore et al. 2014). Table 1 shows that the optimal sample sizes were smaller for *T* than for *W*, and potential errors for *T* at the optimal sample sizes were also smaller than those for *W*. This finding indicated that *W* variation could be a greater source of variability when increasing the scale, which was observed because the experimental site is arid land, and farmland in this area depends on irrigation from the Heihe River. During the growing season, *T* is basically stable. But, *W* is closely related to irrigation, and the irrigation would result in spatial heterogeneity of *W* (Ge et al. 2013; Liu et al. 2015). So in the scaling‐up process, spatial heterogeneity of soil moisture is higher than that of soil temperature.

However, the optimal sample sizes were larger for *R* than for *T* and *W*, and the potential errors for *R* at the optimal sample sizes were larger than those for *T* and similar with those for *W* (Table 1). This suggested that the relationship error of *R* with *W* and/or *T* is not a simple linear relation when increasing the scale. The *R* was based on three models, including *R*:*T*,* R*:*W*, and *R*:*T*&*W*. Although some studies have suggested that biophysiological process (the hysteresis effect, root exudates, photosynthesis and so on) plays an important role in soil respiration, these belong to factors of the process model for soil respiration (Griffis et al. 2003; Gaumont‐Guay et al. 2006; Kuzyakov and Gavrichkova 2010). Actually, the process model is indeed more reliable and rational. Nevertheless, the process model has not been effectively developed and widely used until now (Zhou et al. 2010). In this study, the three functions are classic empirical models and have been confirmed and applied widely. Therefore, the three empirical models were selected for this study.

First, the *R*:*T* model is a more universal *T*‐dependent equation for soil respiration estimation (Lloyd and Taylor 1994). Equation (7) is total derivative form of the *R*:*T* model and indicated that the error of *R* (CV) is linear with variation in *T* (STD). In the scaling‐up process, the constant coefficients (*β*) are stable on the whole when *n *>* *24 (Table 2). Similarly, the *R*:*W* model is also an important equation and, in soil moisture, is a single factor. Equation (8) is a total derivative form of the *R*:*W* model. Equation (8) indicated that the error of *R* is linear with the error of *W*. When *n *>* *24, the constant coefficient (*β*) is stable on the whole in the scaling‐up process. Third, the equation (9) is from the total derivative of *R*:*T*&*W*. This equation suggested that the error of *R* is the multiple of the potential errors in *T* and *W*. Nevertheless, the constant coefficients (*β* and *γ*) are also shown in Table 2. When *n *>* *24, the constant coefficients (*β* and *γ*) are stable on the whole in the scaling‐up process. All above constant coefficients indicate that when the sample size is not large enough, the effect of *T* or *W* on *R* is not steady due to spatial heterogeneity. This demonstrated that if the optimal sample size was obtained from enough sample size in the Monte Carlo method, the error could be significantly reduced. Furthermore, by comparing *β* and *γ* in equation (9), we were able to find that *γ* is more stable than *β* with a sample size change. This also indicated that soil moisture is the dominant factor for soil respiration; variation of steady soil temperature is dependent on strong fluctuation of *β*. In contrast, *W* changes dramatically compared with the variation in *γ*. Besides, Table 3 showed the coefficients of determination (*R* ^2^) of three models on *R* against *T* and/or *W*. The results indicated *W* could explain 55.6 ± 13.2% variation of *R*, but *T* only could explain 28.3 ± 11.6%. Although *R*:*T*&*W* performs the best fitness among three models due to interaction between *T* and *W*, the best fitness of *R*:*T*&*W* was mainly based on *W* factor. That also demonstrated soil respiration is dominated by soil moisture in the field.

For estimating soil CO_2_ emission, choosing an appropriate model is always a difficult problem (Shi et al. 2012); even though Gomez‐Casanovas et al. (2013) evaluated applicability of different models, the evaluation depended on a time series. However, the problem of estimating soil CO_2_ based on spatial heterogeneity has not been solved. The results shown in Figure 4, from use of the Monte Carlo method, show the variation of errors (CV) for *R* based on three models with sample size changes. Apparently, in 51 points in the 5.0 × 5.0 km^2^ plot and 30 points in the 5.0 × 2.5 km^2^ plot, the errors with sample size changes are not different among the three models (Fig. 4A,B). These results indicate that the responses of the three models to sample size change are not different for these point settings. This may because a larger sample is enough to reduce the spatial heterogeneity. However, for 27, 24, and 21 points in the 5.0 × 2.5 km^2^ plot and 15 and 12 points in the 2.5 × 2.5 km^2^, the error of *R*:*T*&*W* is more significantly reduced with sample size change. This means that the *R*:*T*&*W* is more appropriate than other models for these point settings. At these settings, the sample size is not effective for decreasing the spatial heterogeneity from the interaction between *T* and *W*. However, for 9 points in the 2.5 × 2.5 km^2^, *R*:*T* is the best model for reducing error with the scaling‐up process. According to the above analysis, it could be concluded that when the sample size is large enough, the performance of the three models is fine. Nevertheless, a consideration of convenient and traditional applications suggests that *R*:*T* could be an appropriate model. When the sample size is not more than enough, *R*:*T*&*W* would be a better choice. Finally, when the sample size is less for the experimental area, *R*:*T* is also an effective model for increasing accuracy. Furthermore, Table 2 and Fig. 4 indicate that point density (point number/area) may not be an effective proxy for error analysis in comparison with point number.

However, these conclusions are only for this experiment, but it can be demonstrated the spatial Monte Carlo sampling is an effective method or technique for optimizing sample size and filtering model in future studies. These analyses would open a new way to effectively decrease error and shed light on the mechanisms driving soil respiration.

## Conflict of Interest

None declared.

## Supporting information


**Appendix S1.** Preliminary experiment design.

**Table S1.** location of observation points in study area.

## References

[ece31729-bib-0001] Bond‐Lamberty, B. , and A. Thomson . 2010 Temperature‐associated increases in the global soil respiration record. Nature 464:579‐U132. 20336143 10.1038/nature08930

[ece31729-bib-0002] Bond‐Lamberty, B. , C. K. Wang , and S. T. Gower . 2004 A global relationship between the heterotrophic and autotrophic components of soil respiration? Glob. Chang. Biol. 10:1756–1766.

[ece31729-bib-0003] Cramer, W. , A. Bondeau , F. I. Woodward , I. C. Prentice , R. A. Betts , V. Brovkin , et al. 2001 Global response of terrestrial ecosystem structure and function to CO2 and climate change: results from six dynamic global vegetation models. Glob. Chang. Biol. 7:357–373.

[ece31729-bib-0004] Dore, S. , D. L. Fry , and S. L. Stephens . 2014 Spatial heterogeneity of soil CO2 efflux after harvest and prescribed fire in a California mixed conifer forest. For. Ecol. Manage. 319:150–160.

[ece31729-bib-0005] Doucet, A. , S. Godsill , and C. Andrieu . 2000 On sequential Monte Carlo sampling methods for Bayesian filtering. Stat. Comput. 10:197–208.

[ece31729-bib-0006] Elbers, J. A. , C. M. J. Jacobs , B. Kruijt , W. W. P. Jans , and E. J. Moors . 2011 Assessing the uncertainty of estimated annual totals of net ecosystem productivity: a practical approach applied to a mid latitude temperate pine forest. Agric. For. Meteorol. 151:1823–1830.

[ece31729-bib-0007] Field, C. B. , D. B. Lobell , H. A. Peters , and N. R. Chiariello . 2007 Feedbacks of terrestrial ecosystems to climate change Annu. Rev. Env. Resour. 32:1–29.

[ece31729-bib-0008] Gaumont‐Guay, D. , T. A. Black , T. J. Griffis , A. G. Barr , R. S. Jassal , and Z. Nesic . 2006 Interpreting the dependence of soil respiration on soil temperature and water content in a boreal aspen stand. Agric. For. Meteorol. 140:220–235.

[ece31729-bib-0009] Ge, Y. , X. Li , C. Huang , and Z. Nan . 2013 A Decision Support System for irrigation water allocation along the middle reaches of the Heihe River Basin, Northwest China. Environ. Model. Softw. 47:182–192.

[ece31729-bib-0010] Gomez‐Casanovas, N. , K. Anderson‐Teixeira , M. Zeri , C. J. Bernacchi , and E. H. Delucia . 2013 Gap filling strategies and error in estimating annual soil respiration. Glob. Chang. Biol. 19:1941–1952. 23504959 10.1111/gcb.12127

[ece31729-bib-0011] Griffis, T. J. , T. A. Black , K. Morgenstern , et al. 2003 Ecophysiological controls on the carbon balances of three southern boreal forests. Agric. For. Meteorol. 117:53–71.

[ece31729-bib-0012] Hou, Z. S. , D. W. Engel , G. Lin , Y. L. Fang , and Z. F. Fang . 2013 An uncertainty quantification framework for studying the effect of spatial heterogeneity in reservoir permeability on CO2 sequestration. Math. Geosci. 45:799–817.

[ece31729-bib-0013] Huang, X. , Y. N. Chen , W. H. Li , J. X. Ma , and Y. P. Chen . 2007 Daily variation of carbon flux in soils of *Populus euphratica* forests in the middle and lower reaches of the Tarim River. Prog. Nat. Sci. 17:584–590.

[ece31729-bib-0014] Jassal, R. S. , T. A. Black , Z. Nesic , and D. Gaumont‐Guay . 2012 Using automated non‐steady‐state chamber systems for making continuous long‐term measurements of soil CO2 efflux in forest ecosystems. Agric. For. Meteorol. 161:57–65.

[ece31729-bib-0015] Koskinen, M. , K. Minkkinen , P. Ojanen , M. Kamarainen , T. Laurila , and A. Lohila . 2014 Measurements of CO2 exchange with an automated chamber system throughout the year: challenges in measuring night‐time respiration on porous peat soil. Biogeosciences 11:347–363.

[ece31729-bib-0016] Kumagai, T. , S. Aoki , H. Nagasawa , et al. 2005a Effects of tree‐to‐tree and radial variations on sap flow estimates of transpiration in Japanese cedar. Agric. For. Meteorol. 135:110–116.

[ece31729-bib-0017] Kumagai, T. , H. Nagasawa , T. Mabuchi , et al. 2005b Sources of error in estimating stand transpiration using allometric relationships between stem diameter and sapwood area for *Cryptomeria japonica* and *Chamaecyparis obtusa* . For. Ecol. Manage. 206:191–195.

[ece31729-bib-0018] Kume, T. , K. Tsuruta , H. Komatsu , T. Kumagai , N. Higashi , Y. Shinohara , et al. 2010 Effects of sample size on sap flux‐based stand‐scale transpiration estimates. Tree Physiol. 30:129–138. 19822581 10.1093/treephys/tpp074

[ece31729-bib-0019] Kuzyakov, Y. , and O. Gavrichkova . 2010 REVIEW: time lag between photosynthesis and carbon dioxide efflux from soil: a review of mechanisms and controls. Glob. Chang. Biol. 16:3386–3406.

[ece31729-bib-0020] Li, H. J. , J. X. Yan , X. F. Yue , and M. B. Wang . 2008a Significance of soil temperature and moisture for soil respiration in a Chinese mountain area. Agric. For. Meteorol. 148:490–503.

[ece31729-bib-0021] Li, Y. L. , D. Otieno , K. Owen , Y. Zhang , J. Tenhunen , X. Q. Rao , et al. 2008b Temporal variability in soil CO(2) emission in an orchard forest ecosystem. Pedosphere 18:273–283.

[ece31729-bib-0022] Li, X. , G. Cheng , S. Liu , et al. 2013 Heihe watershed allied telemetry experimental research (HiWATER): scientific objectives and experimental design. Bull. Am. Meteorol. Soc. 94:1145–1160.

[ece31729-bib-0023] Liu, H. , W. Z. Zhao , Z. B. He , and J. T. Liu . 2015 Soil moisture dynamics across landscape types in an arid inland river basin of Northwest China. Hydrol. Process. 29:3328–3341.

[ece31729-bib-0024] Lloyd, J. , and J. A. Taylor . 1994 On the temperature‐dependence of soil respiration. Funct. Ecol. 8:315–323.

[ece31729-bib-0025] Maier, M. , and H. Schack‐Kirchner . 2014 Using the gradient method to determine soil gas flux: a review. Agric. For. Meteorol. 192:78–95.

[ece31729-bib-0026] Oren, R. , C. I. Hseih , P. Stoy , J. Albertson , H. R. Mccarthy , P. Harrell , et al. 2006 Estimating the uncertainty in annual net ecosystem carbon exchange: spatial variation in turbulent fluxes and sampling errors in eddy‐covariance measurements. Glob. Chang. Biol. 12:883–896.

[ece31729-bib-0027] Piao, S. L. , J. Y. Fang , P. Ciais , P. Peylin , Y. Huang , S. Sitch , et al. 2009 The carbon balance of terrestrial ecosystems in China. Nature 458:1009‐U1082. 19396142 10.1038/nature07944

[ece31729-bib-0028] Pumpanen, J. , P. Kolari , H. Ilvesniemi , et al. 2004 Comparison of different chamber techniques for measuring soil CO2 efflux. Agric. For. Meteorol. 123:159–176.

[ece31729-bib-0029] Raich, J. W. , and C. S. Potter . 1995 Global patterns of carbon‐dioxide emissions from soils. Global Biogeochem. Cycles 9:23–36.

[ece31729-bib-0030] Reichstein, M. , A. Rey , A. Freibauer , et al. 2003 Modeling temporal and large‐scale spatial variability of soil respiration from soil water availability, temperature and vegetation productivity indices. Global Biogeochem. Cycles 17:1104.

[ece31729-bib-0031] Richardson, A. D. , and D. Y. Hollinger . 2007 A method to estimate the additional uncertainty in gap‐filled NEE resulting from long gaps in the CO_2_ flux record. Agric. For. Meteorol. 147:199–208.

[ece31729-bib-0032] Riederer, M. , A. Serafimovich , and T. Foken . 2014 Net ecosystem CO_2_ exchange measurements by the closed chamber method and the eddy covariance technique and their dependence on atmospheric conditions. Atmos. Meas. Tech. 7:1057–1064.

[ece31729-bib-0033] Schlesinger, W. H. , and J. A. Andrews . 2000 Soil respiration and the global carbon cycle. Biogeochemistry 48:7–20.

[ece31729-bib-0034] Shi, W. Y. , R. Tateno , J. G. Zhang , Y. L. Wang , N. Yamanaka , and S. Du . 2011 Response of soil respiration to precipitation during the dry season in two typical forest stands in the forest‐grassland transition zone of the Loess Plateau. Agric. For. Meteorol. 151:854–863.

[ece31729-bib-0035] Shi, W. Y. , J. G. Zhang , M. J. Yan , N. Yamanaka , and S. Du . 2012 Seasonal and diurnal dynamics of soil respiration fluxes in two typical forests on the semiarid Loess Plateau of China: temperature sensitivities of autotrophs and heterotrophs and analyses of integrated driving factors. Soil Biol. Biochem. 52:99–107.

[ece31729-bib-0036] Shi, W.‐Y. , M.‐J. Yan , J.‐G. Zhang , J.‐H. Guan , and S. Du . 2014 Soil CO_2_ emissions from five different types of land use on the semiarid Loess Plateau of China, with emphasis on the contribution of winter soil respiration. Atmos. Environ. 88:74–82.

[ece31729-bib-0037] Subke, J. A. , I. Inglima , and M. F. Cotrufo . 2006 Trends and methodological impacts in soil CO(2) efflux partitioning: a metaanalytical review. Glob. Chang. Biol. 12:921–943.

[ece31729-bib-0038] Trumbore, S. 2006 Carbon respired by terrestrial ecosystems ‐ recent progress and challenges. Glob. Chang. Biol. 12:141–153.

[ece31729-bib-0039] Vargas, R. , M. S. Carbone , M. Reichstein , and D. D. Baldocchi . 2011 Frontiers and challenges in soil respiration research: from measurements to model‐data integration. Biogeochemistry 102:1–13.

[ece31729-bib-0040] Wang, H. , B. Mcconkey , D. Curtin , and H. Cutforth . 2010a Estimation of daily soil CO_2_ flux using a single‐time‐point measurement. Can. J. Soil Sci. 90:517–522.

[ece31729-bib-0041] Wang, W. , S. S. Peng , T. Wang , and J. Y. Fang . 2010b Winter soil CO_2_ efflux and its contribution to annual soil respiration in different ecosystems of a forest‐steppe ecotone, north China. Soil Biol. Biochem. 42:451–458.

[ece31729-bib-0042] Wang, J. , X. Li , L. Lu , and F. Fang . 2013 Estimating near future regional corn yields by integrating multi‐source observations into a crop growth model. Eur. J. Agron. 49:126–140.

[ece31729-bib-0043] Xu, M. , and Y. Qi . 2001 Soil‐surface CO_2_ efflux and its spatial and temporal variations in a young ponderosa pine plantation in northern California. Glob. Chang. Biol. 7:667–677.

[ece31729-bib-0044] Yu, G. R. , Z. M. Zheng , Q. F. Wang , Y. L. Fu , J. Zhuang , X. M. Sun , et al. 2010 Spatiotemporal pattern of soil respiration of terrestrial ecosystems in China: the development of a geostatistical model and its simulation. Environ. Sci. Technol. 44:6074–6080. 20704202 10.1021/es100979s

[ece31729-bib-0045] Zhang, Q. , H. M. Lei , and D. W. Yang . 2013 Seasonal variations in soil respiration, heterotrophic respiration and autotrophic respiration of a wheat and maize rotation cropland in the North China Plain. Agric. For. Meteorol. 180:34–43.

[ece31729-bib-0046] Zhou, X. H. , Y. Q. Luo , C. Gao , P. S. J. Verburg , J. A. Arnone , A. Darrouzet‐Nardi , et al. 2010 Concurrent and lagged impacts of an anomalously warm year on autotrophic and heterotrophic components of soil respiration: a deconvolution analysis. New Phytol. 187:184–198. 20412445 10.1111/j.1469-8137.2010.03256.x

