# Supplementary material for: A Monte Carlo approach to estimate the uncertainty in soil CO2 emissions caused by spatial and sample size variability
Source: Ecol Evol. 2015 Sep 23;5(19):4480–91. doi: 10.1002/ece3.1729 (PMC4667816; doi:10.1002/ece3.1729)
Supplement: Supplementary file 1 — Appendix S1. Preliminary experiment design. [file ECE3-5-4480-s001.doc]

**Appendix S1:** Preliminary experiment design

A PVC collar was installed in the same plot for continuous measurement in 2011. Soil respiration was automatically continuously measured (Li-8100-103, Li-COR, USA) from19 June to 31 August, 2011 and the data was recorded every 30 min. this collar was named “C point”.

An automated weather station (AWS) was installed in April, 2011 adjoining C point. The AWS is continuously running as a long-term observation station. Environmental data sampled included air temperature and relative humidity measured by a thermohygrograph (AV-14TH, Avalon, USA), and precipitation measured by a tipping bucket rain gauge (TE525MM, TEXAS ELECTRONICS, USA). Soil temperature was measured at 0, 2, 4, 10, 20 and 40 cm depth by thermo recorders (AV-10T, Avalon, USA), and soil moisture was measured at 2, 4, 10, 20 and 40 cm by a soil moisture sensor (CS616, Campbell, USA). Precipitation was recorded following each event. All continuous measurements were performed at 30 s intervals, and 30-min averages were recorded.
