# Supplementary material for: A Monte Carlo approach to estimate the uncertainty in soil CO2 emissions caused by spatial and sample size variability
Source: Ecol Evol. 2015 Sep 23;5(19):4480–91. doi: 10.1002/ece3.1729 (PMC4667816; doi:10.1002/ece3.1729)
Supplement: Supplementary file 2 — Table S1. location of observation points in study area. [file ECE3-5-4480-s002.doc]

Table S1 location of observation points in study area

| Subplot | Point | longitude | latitude |
| --- | --- | --- | --- |
| Subplot 1 | 1 | 100.358163° | 38.893218° |
|  | 2 | 100.358165° | 38.893216° |
|  | 3 | 100.358167° | 38.893214° |
|  | 4 | 100.354065° | 38.886973° |
|  | 5 | 100.354067° | 38.886971° |
|  | 6 | 100.354069° | 38.886969° |
|  | 7 | 100.376325° | 38.890566° |
|  | 8 | 100.376323° | 38.890564° |
|  | 9 | 100.376321° | 38.890562° |
|  | 10 | 100.357538° | 38.877546° |
|  | 11 | 100.357536° | 38.877548° |
|  | 12 | 100.357534° | 38.877550° |
|  | 13 | 100.365230° | 38.876768° |
|  | 14 | 100.365229° | 38.876771° |
|  | 15 | 100.365228° | 38.876774° |
| Subplot 2 | 16 | 100.376467° | 38.872548° |
|  | 17 | 100.376469° | 38.872546° |
|  | 18 | 100.376471° | 38.872544° |
|  | 19 | 100.385477° | 38.872407° |
|  | 20 | 100.385475° | 38.872409° |
|  | 21 | 100.385473° | 38.872411° |
|  | 22 | 100.395702° | 38.875693° |
|  | 23 | 100.395700° | 38.875695° |
|  | 24 | 100.395698° | 38.875697° |
|  | 25 | 100.366329° | 38.865158° |
|  | 26 | 100.366331° | 38.865156° |
|  | 27 | 100.366333° | 38.865154° |
|  | 28 | 100.378489° | 38.860767° |
|  | 29 | 100.378487° | 38.860765° |
|  | 30 | 100.378485° | 38.860763° |
| Subplot 3 | 31 | 100.350649° | 38.875772° |
|  | 32 | 100.350647° | 38.875770° |
|  | 33 | 100.350645° | 38.875768° |
|  | 34 | 100.359723° | 38.871200° |
|  | 35 | 100.359721° | 38.871198° |
|  | 36 | 100.359719° | 38.871196° |
|  | 37 | 100.341978° | 38.869936° |
|  | 38 | 100.341976° | 38.869934° |
|  | 39 | 100.341974° | 38.869932° |
|  | 40 | 100.353120° | 38.858685° |
|  | 41 | 100.353118° | 38.858687° |
|  | 42 | 100.353116° | 38.858689° |
| Subplot 4 | 43 | 100.372265° | 38.855553° |
|  | 44 | 100.372262° | 38.855553° |
|  | 45 | 100.372259° | 38.855553° |
|  | 46 | 100.364134° | 38.849303° |
|  | 47 | 100.364133° | 38.849305° |
|  | 48 | 100.364132° | 38.849307° |
|  | 49 | 100.369744° | 38.845119° |
|  | 50 | 100.369744° | 38.845121° |
|  | 51 | 100.369744° | 38.845123° |
